# Identification of *Naegleria fowleri* antigens recognized by serum antibodies from people of Mexicali Valley, México

**DOI:** 10.1007/s00436-025-08476-2

**Published:** 2025-03-15

**Authors:** Itzel Berenice Rodríguez-Mera, Saúl Rojas-Hernández, Patricia Bonilla-Lemus, Mariela Esquivel-Solís, Frida Carrillo-Morales, Mara Gutiérrez-Sánchez, Israel López-Reyes, José Luis Osornio-Rojas, María Maricela Carrasco-Yépez

**Affiliations:** 1 Laboratorio de Microbiología Ambiental, Grupo CyMA, UIICSE, FES Iztacala, Universidad Nacional Autónoma de México, Estado de México, Tlalnepantla de Baz, México; 2 Laboratorio de Inmunología Molecular y de Mucosas, Escuela Superior de Medicina, Instituto Politécnico Nacional, Ciudad de Mexico, México; 3 Universidad Autónoma de La Ciudad de México (UACM), Plantel Cuautepec, Av. La Corona 320, Col. Loma La Palma, Alcaldía Gustavo A. Madero, C.P. 07160, Ciudad de Mexico, México; 4 Departamento de Estomatología, Universidas Autónoma de Ciudad Juarez, Ciudad Juárez, Chihuahua México

**Keywords:** *Naegleria fowleri*, PAM, Mexicali Valley

## Abstract

*Naegleria fowleri* is an amoeba that causes a fatal disease in the central nervous system known as primary amoebic meningoencephalitis (PAM) in humans. Most of the infections are acquired by people who practice recreational activities in water contaminated with trophozoites. Swimming and wading in irrigation channels of Mexicali are common practices for local people. Although there are some warning signposts in the surrounding sites, people continue using these channels for recreational purposes. In that region, cases of PAM have been reported; however, not everyone who comes into contact with contaminated water containing trophozoites becomes infected, and the factors influencing their immune response to *N. fowleri* remain unknown. We analyzed the levels of antibodies against *N. fowleri* in two groups: local individuals, including visitors who swam in the Mexicali channels, and a group from Mexico City (CDMX). In both groups, specific antibody responses were analyzed using immunoassays, including Western blot, ELISA, and cytochemistry. The highest levels of both IgG and IgA were found in samples from Mexicali, compared to those from CDMX. In both groups, IgG recognized polypeptide bands from *N. fowleri* at molecular weights of 100, 50, and 19 kDa, bands that we have already reported as immunogenic. Moreover, the IgG subjects recognized trophozoite structures such as membrane, pseudopodia, food cups, and even small like-vesicles. This antibody immune response directed against these polypeptide bands and trophozoite structures along with other factors could be participating in the defense of these people against PAM.

**Supplementary Information:**

The online version contains supplementary material available at 10.1007/s00436-025-08476-2.

## Introduction

Protozoa infections are the main causes of death worldwide (Martínez-Castillo et al. 2016; Mishra et al. 2009). These include a group of opportunistic microorganisms known as free-living amoebae (FLA) which can cause serious health problems. *Acanthamoeba*, *Balamuthia*, and *Naegleria* are some of the main FLA capable of causing fatal infections in humans (Siddiqui et al. 2016; Visvesvara 2013; Visvesvara, Moura, & Schuster, 2007).

*Naegleria fowleri* is a free-living thermophilic amoeboflagellate that causes primary amoebic meningoencephalitis (PAM), a disease of the central nervous system (CNS) that begins when trophozoites enter the nasal cavity and invade the nasal mucosa. Subsequently, the trophozoites adhere to the epithelium and migrate along the olfactory nerves through the cribriform plate to access the olfactory bulb and brain (Aurongzeb et al. 2021; Moseman 2020; Rojas-Hernández et al. 2004). PAM is a fatal infection with a global distribution that mainly affects healthy children and young adults with aquatic activities history in natural or artificial freshwater bodies (Dzikowiec et al. 2017). Symptoms are like a viral or bacterial meningitis which includes fever, nausea, vomiting, headache, irritability, neck stiffness, and in more severe cases seizures (Ur Rehman et al. 2024). The abovementioned, combined with the rapid progress of the infection, lead to host death in a period of 3–7 days after the initial exposure, causing high mortality percentages (95–99%) (Dzikowiec et al. 2017; Jahangeer et al. 2020).

Although this disease is rare, new cases are reported every year around the world (Cope & Ali 2016; Gompf & Garcia 2019; Yoder et al. 2010; Zhang et al. 2018). The first case of PAM was described in Australia in 1965 (Fowler & Carter 1965). Since then, several reviews have mentioned that an estimated 400 cases have been reported worldwide (Jahangeer et al. 2020; Maciver et al. 2020; Nadeem et al. 2023).

According to recent data, a total of 39 countries have reported cases of PAM, being the USA, Pakistan, México, India, Australia, and the Czech Republic the most affected (Jahangeer et al. 2020; Nadeem et al. 2023). In Mexico, under scientific rigor, 11 cases of PAM have been documented (Cervantes-Sandoval et al. 2007; Lares-Villa et al. 1993; López-Corella et al. 1989; Valenzuela et al. 1984; Vargas-Zepeda et al. 2005), while in local epidemiological alerts, around 30 cases have been described (Gharpure et al. 2021), leading México to occupy third place in the number of reported cases. It is important to highlight that, due to the lack of knowledge of this disease, as well as its incidence and prevalence, there may be misdiagnosed cases causing a number of variations of reported cases (Matanock et al. 2018; Zhang et al. 2018).

Mexicali Valley in Baja California has an intensive agricultural system that depends on an irrigation system made up of a channel network fed by the Colorado River (Bonilla-Lemus et al. 2020). The Valley is characterized by a desert climate and high temperatures prevalence mainly in the summer months, causing irrigation channels to be used for aquatic activities (Lares-Villa et al. 1993). Previously, PAM cases have been reported in people with aquatic activity history in these channels (Cervantes-Sandoval et al. 2007; Lares-Villa et al. 1993) as well as the presence of *Naegleria* spp. in water samples collected from different channels in Mexicali Valley (Bonilla-Lemus et al. 2020; Lares-Villa et al. 1993).

Identifying immunogenic antigens from *N. fowleri* is crucial for developing vaccines and diagnostic tools. This study assessed the IgA and IgG antibody responses as well as the specific recognition of *N*. *fowleri* polypeptide bands by IgG in individuals with varying levels of exposure to the amoeba. Participants included residents from both endemic areas, like the Mexicali Valley, and non-endemic regions, such as Mexico City (CDMX).

## Methodology

### Ethics statement

This research was conducted in accordance with the Helsinki Declaration, and the work protocol was reviewed and approved by the Institutional Ethical Committee of the Faculty of Higher Studies Iztacala, UNAM (Number of Approval CE/FESI/022020/1317). In addition, prior to our study, we obtained informed consent from all participants in this study. In the case of children under 10 years of age, consent was requested from their guardians.

**Fig. 1:**
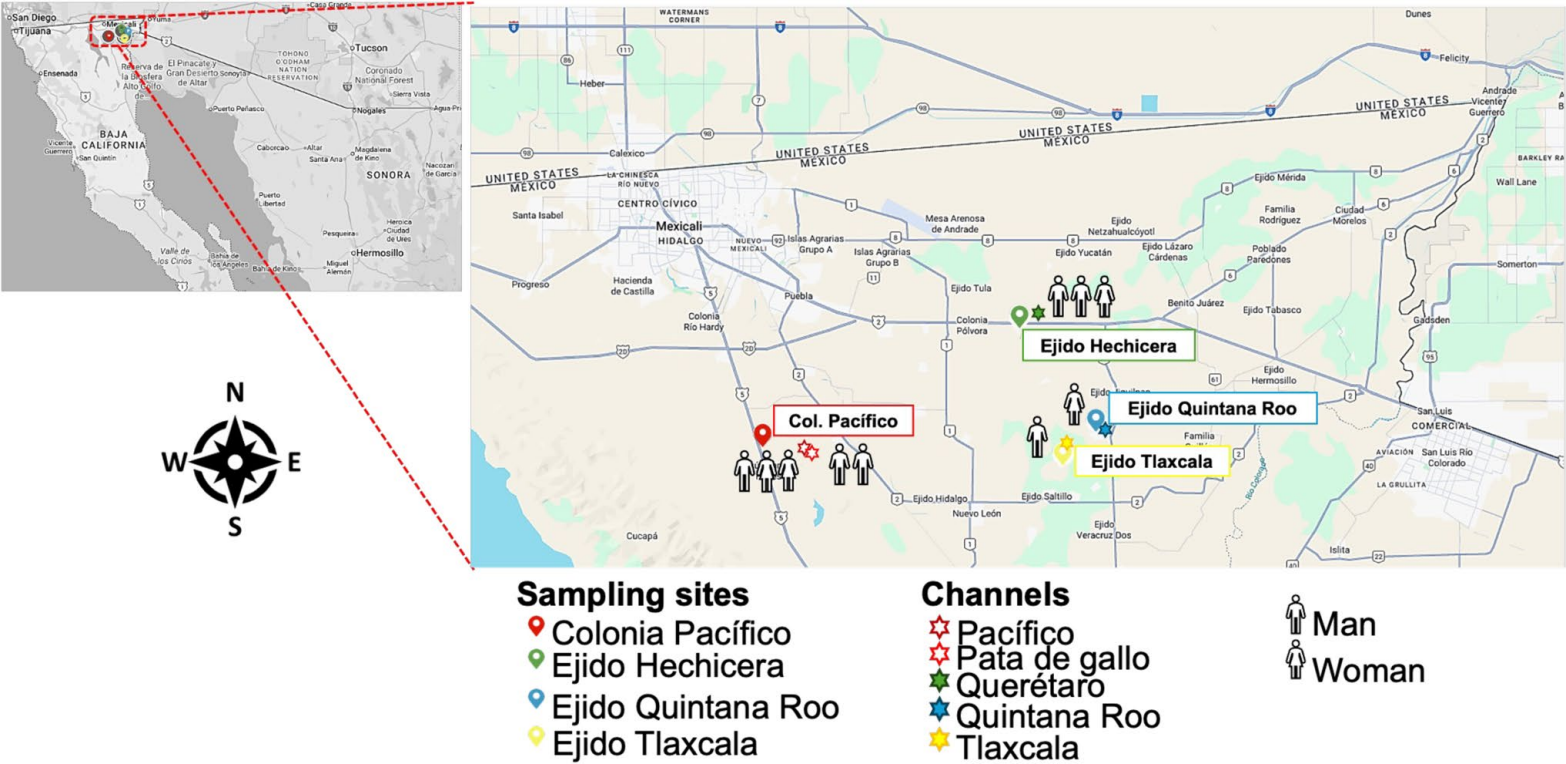
Map of the Mexicali Valley in northern Mexico. The map displays sampling sites marked with colored symbols, while irrigation channels related to water activities in the area are indicated by colored stars. The number of samples collected and the gender of the participants are represented by male and female figures on the map. This is a modified version of a map from Google Maps

A cross-sectional study was conducted using a single serum sample from each participant to assess the levels and recognition of antibodies against *N. fowleri* antigens. All samples were collected in July 2019 from individuals living in three different shared lands, or ejidos (Fig. 1) in the Mexicali Valley, as well as participants from a laboratory where staff work with *N. fowleri* in Mexico City (CDMX) who were invited to voluntarily participate in this study.

### Sample collection

Approximately 5 mL of blood was obtained by vein puncture in Vacutainer tubes of a total of 15 volunteers including children, adolescents, and adults, both women and men. A total of 10 samples were collected in three ejidos in the Mexicali Valley, Mexico, and 5 from CDMX residents. In the case of the Mexicali samples, it was considered that the subjects were doing some recreational aquatic activity at the time of sampling. Each sample was accompanied by host data such as sex, age, activity in Mexicali channels, and frequency of use (Table 1). For serum collection of each subject, tubes were centrifuged at 1500 × g, 20 min at 4 °C, aliquoted in plastic tubes, and stored at − 80 °C until analysis.

**Table 1:** General sociodemographic data of serum sample donors from both Mexicali and CDMX

Sample	Sex	Age	Activity in Mexicali canals		History of amoebiasis
			Domestic (every day)	Recreational (mainly in summer)	
Mexicali samples					
M1	W	10	X	-	No
M2	M	52	-	X	No
M3	M	43	-	X	No
M4	W	13	-	X	No
M5	W	41	X	-	No
M6	M	15	X	X	No
M7	M	19	X	X	No
M8	W	27	X	X	No
M9	M	17	X	X	No
M10	M	35	X	X	No
CDMX samples					
L1	M	53	-	-	No
L2	M	32	-	-	No
L3	M	31	-	-	No
L4	W	25	-	-	No
L5	M	30	-	-	No

M1–M10, Mexicali samples; L1–L5, CDMX samples; W, woman; M, man; X, perform that activity; -, does not carry out that activity

### Naegleria fowleri culture

*Naegleria fowleri* ATCC 30808 trophozoites were cultured under axenic conditions at 37 °C in Bactocasitone medium (Gibco) supplemented with 10% fetal bovine serum (BSA) (Gibco). The trophozoites were cooled and harvested in the logarithmic phase of growth (48 H), after centrifugation at 1500 × g for 10 min, the pellets were resuspended in 2 mL of p-hydroxymercuribenzoic acid as a protease inhibitor and stored at − 70 °C until use. The amoebic lysate was performed by continuous sonication cycles for 5 min at 100-W amplitude (BRANSON Digital Sonifier, Model S-150D).

### Enzyme-linked immunosorbent assay

The specific antibody levels against *N. fowleri* in serum samples from subjects from Mexicali and CDMX were determined by the ELISA technique. Briefly, 96-well plates were coated with 10 μg of *N. fowleri* amoebic lysate in carbonate buffer (15 mmol L^−1^ Na2CO_3_, 35 mmol L^−1^ NaHCO3 (pH 9.6)) and incubated for 24 h at 4 °C. The plates were blocked with 6% low-fat milk in PBS-T. Subsequently, 1:100 of each serum sample was added to 100 μL of PBS-T. For the pools, 10 μL of each sample was mixed and added in a 1:100 dilution. After 24 h of incubation at 4 °C, the goat anti-human IgG or IgA conjugated horseradish peroxidase-labelled was added as secondary antibody (1:1000 or 1:250 respectively) in 100 μL PBS-T. Between each treatment, the plates were washed three times with PBS-T.

### Electroelution of 100, 50, and 19 kDa bands from Naegleria fowleri

A total of 20 µg of *N. fowleri* amoebic lysate was separated by SDS-PAGE (14%) under reducing conditions at 100 V. Subsequently, 16 gels were stained with Coomassie blue to identify the 100, 50, and 19 kDa bands. Once each band was identified, they were cut and kept separately in elution buffer (25 mM Tris base, 192 mM glycine, and 0.1% SDS) and stored at 4 °C until use. The cut bands for each molecular weight were electroeluted with an Electro-eluter (Model 422) at a voltage of 60 mA for 5 h. The samples obtained were dialyzed overnight at 4 °C in PBS-1 × , after which the samples were recovered and concentrated by centrifugation cycles at 600 × g until obtaining an approximate volume of 500 μL. The proteins obtained were quantified by the Bradford method and examined by 14% SDS-PAGE.

### Immunoblot

Twenty micrograms of *N. fowleri* amoebic lysate or 10 µg of each electroeluted band was separated by SDS-PAGE (14%) and analyzed by immunoblot. Briefly, proteins were separated on polyacrylamide gels and transferred to nitrocellulose membranes. The membranes were blocked with 10% low-fat milk overnight at 4 °C. All membranes were incubated with human sera (1:100) in PBS-T. After 24 h of incubation at 4 °C, the membranes were incubated with goat anti-human IgG HRP (Pierce, 31,413) or goat anti-human IgA HRP (PA174395) (1:1000 and 1:500 respectively) overnight at 4 °C. The protein recognition pattern was revealed with 4-chloro-1-naphtol (Thermo Scientific) and stopped with PBS-T.

### Immunocytochemistry

*N. fowleri* trophozoites (1 × ${10}^{6}$ cells/mL) were incubated on glass coverslips in 24-well plates for 1 h at 37 °C. After the incubation time, the samples were fixed with 2% paraformaldehyde for 30 min at 37 °C and blocked with 1% BSA. All samples were permeabilized with PBS-TT (10 mM NaH2PO4, 150 mM NaCl, 0.005% Tween-20 and 0.5% Triton, pH 7.2) for 5 min at room temperature. For immunodetection, samples were incubated with rabbit anti-*N. fowleri* and human sera (1:100) overnight at 4 °C. Then, samples were washed three times with PBS and incubated with donkey anti-rabbit IgG Alexa Fluor 647 (A31573) and goat anti-human IgG, FITC (ab6854) (1:1000) overnight at 4 °C. All samples were counterstained with 4,6-diamidino-2-phenylindole (DAPI) for nuclei detection and mounted with VECTASHIELD (Vector Laboratories, Inc.). Images were obtained and analyzed with an Axioscop 2 mot plus confocal fluorescence microscope (Carl Zeiss).

### Statistical analysis

Data represents the mean ± SD of three independent trials. Multiple comparisons between the levels of each isotype of Mexicali vs CDMX were performed using a two-way ANOVA with *p* < 0.05. Analysis was performed with Prism GraphPad v 10 statistical software.

## Results

### Specific response of IgG and IgA antibodies in serum samples

**Fig. 2:**
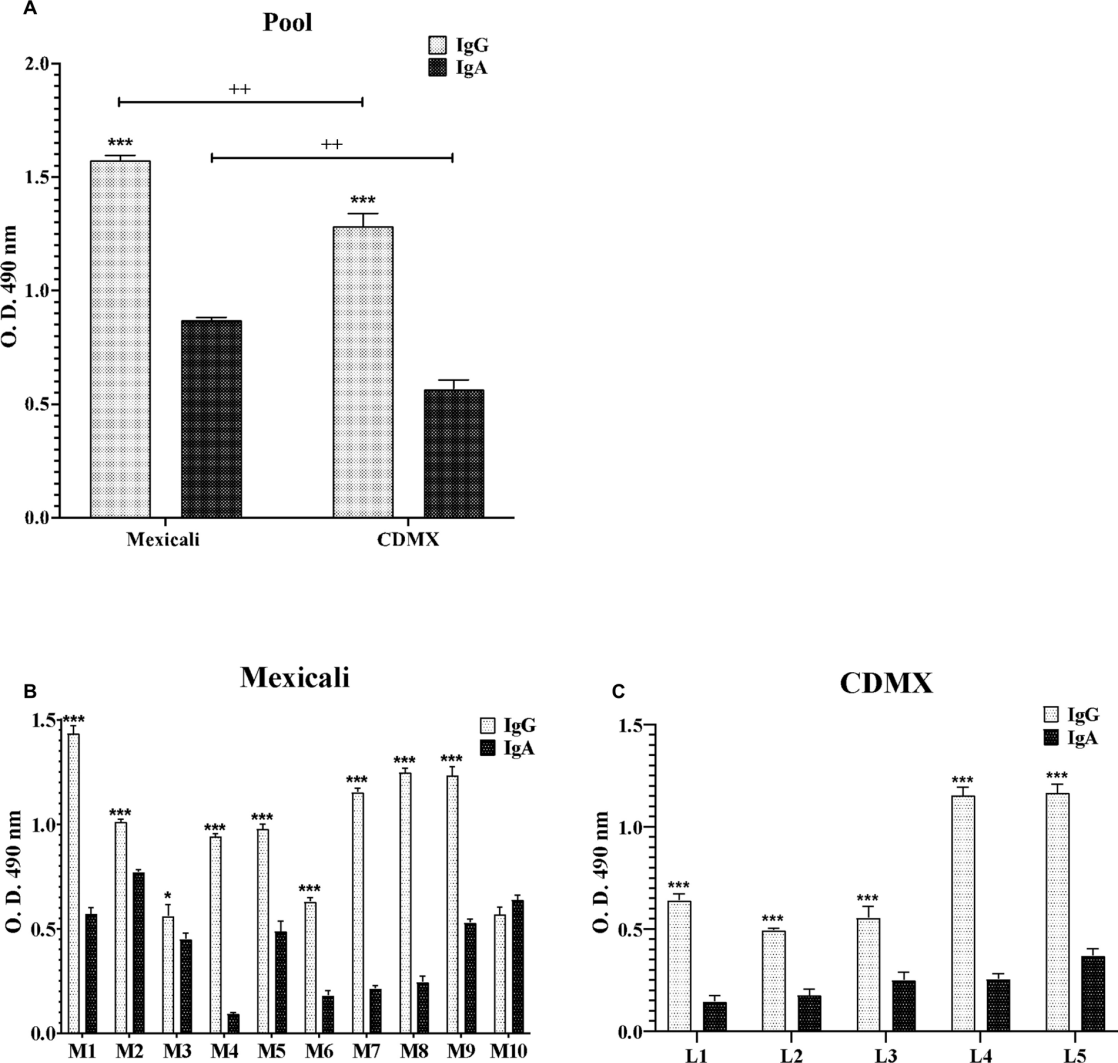
Specific response of IgG and IgA antibodies in serum samples. IgG and IgA antibodies were quantified by the ELISA technique using *N. fowleri* lysate and pooled serum samples (**A**) or per individual (**B** and **C**) obtained from Mexicali Valley or CDMX residents. All plates were sensitized with *N. fowleri* lysate and incubated with 10 samples from Mexicali (**B**, M1–M10) or with 5 samples from CDMX (**C**, L1–L5). Data represents the mean ± SD of three independent trials. Significant differences in the levels of each isotype (IgG vs IgA) are indicated as follows: *p < 0.05, **p < 0.01 ***p < 0.001

Antibody levels present in serum samples from Mexicali Valley and CDMX subjects were evaluated by the ELISA technique. In both subject groups, high levels of specific antibodies against *N. fowleri* were found (Fig. 2). Firstly, we analyzed the pooling serum samples for each group (Fig. 2A) where we can observe that the highest levels for both IgG and IgA were found in Mexicali samples compared to those levels found in samples from CDMX subjects (Fig. 2A, *p* < 0.01**). In addition, in both populations, serum IgG levels were significantly higher with respect to IgA (*p* < 0.001***).

When the antibody levels were analyzed per individual sample (Fig. 2B and C), we also observed most of the Mexicali subjects had the highest IgG and IgA antibody levels.

The Mexicali samples with the highest levels of IgG were M1, M2, M4, M5, M7, M8, and M9 (Fig. 2B), while for CDMX, L4 and L5 samples had the highest levels of IgG (Fig. 2C). Regarding Mexicali serum, samples with the highest levels of IgA were M1, M2, M3, M5, M9, and M10, while for CDMX, no statistically significant differences were found with respect to the levels of serum IgA. Likewise, the M1 sample had the highest levels of IgG while the M3 sample showed the lowest levels. For IgA levels, the M2 sample had the highest levels compared to M4 in which the lowest levels were observed (Fig. 2B). Regarding CDMX samples, L4 and L5 were those samples that showed the highest levels of IgG while the levels of IgA antibodies remained similar for the subjects of this group (Fig. 2C).

### Recognition of polypeptide bands from N. fowleri by IgG e IgA from serum samples

**Fig. 3:**
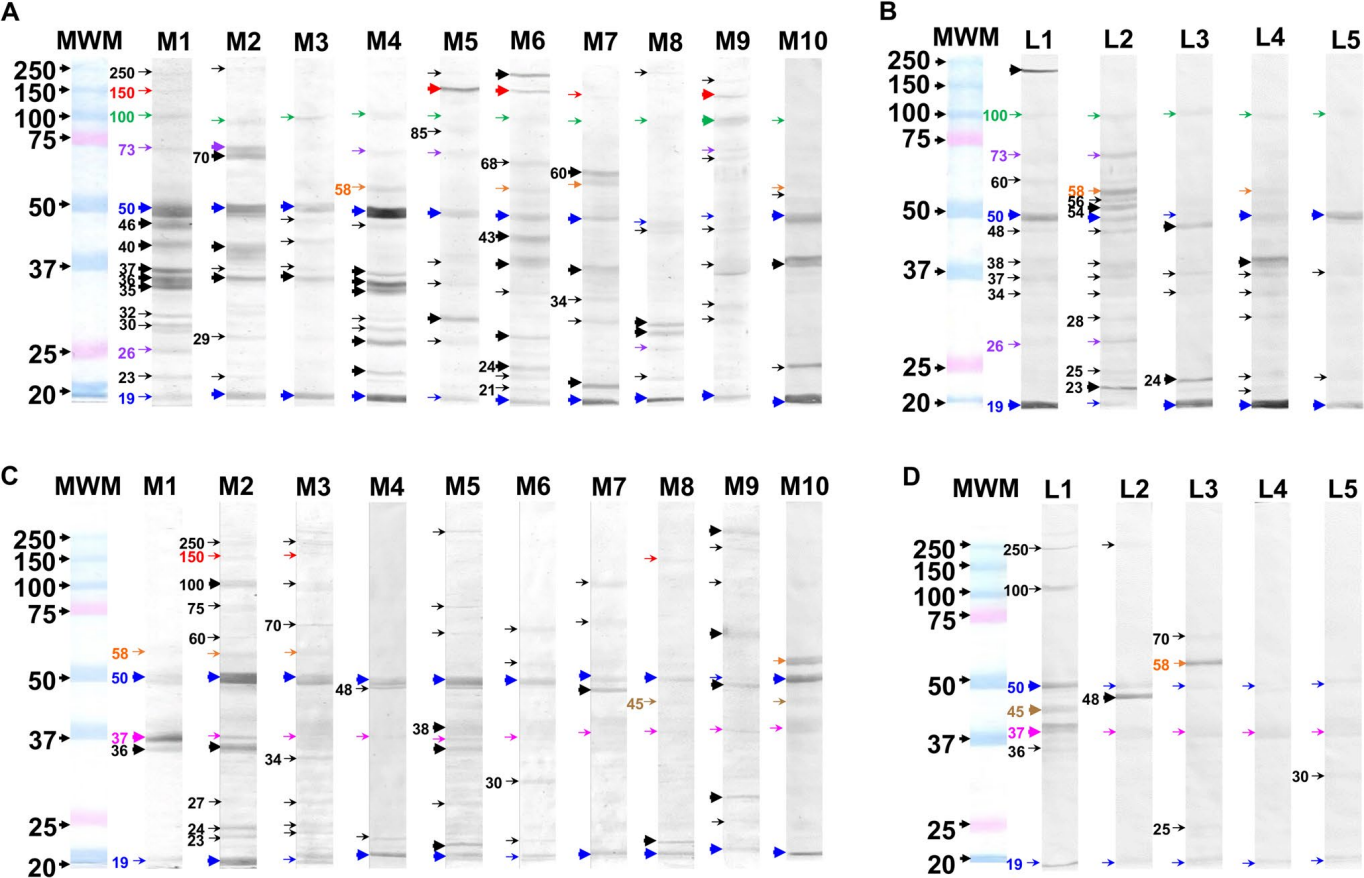
Recognition of polypeptide bands from *N. fowleri* by IgG from serum samples. *N. fowleri* lysates were subjected to SDS-PAGE electrophoresis and transferred to nitrocellulose membranes. The membranes were incubated with serum from Mexicali Valley (**A** and **C**) or CDMX (**B** and **D**) residents and goat anti-human IgG HRP (**A** and **B**) or goat anti-human IgA HRP (**C** and **D**). The numbers on the left represent the molecular weight in kDa. The color code on the arrows represents: blue arrows, antigens present on 50 and 19 kDa polypeptides recognized by both IgG and IgA isotypes in all samples; green arrows, antigens present on 100 kDa polypeptide recognized only by the IgG isotype in all samples; pink arrows, antigens present on 37 kDa polypeptide recognized only by the IgA isotype in all samples; orange arrows, antigens present on 58 kDa polypeptide that were recognized by both isotypes but not in all samples; purple arrows, antigens recognized on 73 and 26 kDa polypeptides recognized by IgG only in some samples; brown arrows, antigens recognized in 45 kDa polypeptide recognized by IgA only in some samples and red arrows, antigens present in the 150 kDa polypeptide recognized by both isotypes only in the Mexicali samples. Molecular weight marker (MWM)

Due to the high levels of specific IgG antibodies against *N. fowleri* present in all serum samples in both subjects of Mexicali and CDMX, the recognition of immunogenic antigens of *N. fowleri* was evaluated by the immunoblot technique (Fig. 3). In general, both IgG and IgA antibodies from all serum samples evaluated recognized antigens present in *N. fowleri* lysate. This antibody recognition included polypeptide bands with molecular weight from 19 to 250 kDa. In both populations (Mexicali and CDMX), the isotype with the highest polypeptide recognition was IgG with approximately 31 different polypeptide bands (Fig. 3A and B) compared to IgA which recognized approximately 20 different polypeptide bands (Fig. 3C, D). Interestingly, the Mexicali samples (Fig. 3A and C) exhibited the highest number of polypeptides recognized by both isotypes compared to the polypeptide bands recognized by both isotypes from subjects in CDMX (Fig. 3B and D). This immunoblot result suggests that the immunological response detected through the high levels of IgG (Fig. 2) could be directed mainly to determined polypeptide bands which are being recognized with different intensities by the antibodies. For example, the IgG of the M1 Mexicali subject recognized approximately 15 polypeptide bands of *N. fowleri* with a relative molecular weight (rMW) from 19 to 250 kDa; however, the bands that were observed with stronger labeling were those with rMW of 35, 36, 37, 40, 46, and 50 kDa (Fig. 3A, thick arrows). It is important to mention that in this case, the same subject had the highest levels of IgG detected by ELISA (Fig. 2B). The findings from subjects M8 and M9 in Mexicali, who also displayed high antibody levels as detected by ELISA, contrast with the results observed in subject M1. In these cases, the antibodies recognized a pattern of polypeptide bands that is less intense and includes fewer bands overall. In the same subjects, certain bands are gaining recognition at varying levels of intensity, with their rMW primarily corresponding to polypeptide bands of 19, 30, 32, 100, and 150 kDa (Fig. 3A).

Another recognition pattern considered interesting due to the consistency between the ELISA (Fig. 2B) and western blot was found by the IgA of the M2 Mexicali subject which mainly recognized 13 polypeptide bands from *N. fowleri* and some of them with a rMW of 19, 36, 50, and 100 kDa were recognized with a strong intensity (Fig. 3C, thick arrows).

Regarding the CDMX samples, the IgG from the L2 subject recognized approximately 15 polypeptide bands with a rMW from 19 to 100 kDa (Fig. 3B), being the polypeptides of 23, 50, 54, and 58 kDa those that had the highest labeling intensity. Even though this subject showed a wide recognition of polypeptide bands through the IgG antibody, the antibody levels detected by ELISA were among the lowest (Fig. 2C). Finally, the IgA from the L1 CDMX subject recognized 7 polypeptide bands whose bands that were observed with stronger labeling were those with a rMW of 37, 45, and 50 kDa (Fig. 3D). The serum IgA from the other subjects in this group (L2, L3, L4, and L5) recognized a smaller number of bands. However, the polypeptide bands that showed particularly strong recognition by the IgA were those at 30, 48, 50, 58, and 70 kDa (Fig. 2D).

It is important to note that the 19 kDa and 50 kDa polypeptide bands were recognized with varying intensity by both isotypes (IgG and IgA) across all evaluated samples (Fig. 3, blue arrows). Notably, the Mexicali group exhibited the strongest recognition in terms of intensity. In addition, it could also be observed that the IgG of all subjects from both groups (Mexicali and CDMX) recognized the 100 kDa polypeptide band with a lower intensity (Fig. 3A and B, green arrows), while the IgA from all subjects (both groups) recognized the 37 kDa polypeptide (Fig. 3C and D, pink arrows).

It was also found that the 58 kDa polypeptide was recognized by both isotypes, but only in specific samples (Fig. 3, orange arrows). In addition, the 26 and 73 kDa polypeptides were only recognized by IgG (Fig. 3A and B, purple arrows) while the 45 kDa was only recognized by IgA (Fig. 3C and D, brown arrows), and the 150 kDa was recognized by both isotypes, but only in the Mexicali samples (Fig. 3A and C, red arrows).

Finally, the samples with the highest number of polypeptide bands and intensity recognized by the IgG isotype were M1, M2, M4, and M10 for Mexicali and L1, L2, and L4 for CDMX, while for IgA, the samples were M2, M5, and M10 for Mexicali and L1, L2, and L3 for CDMX (Fig. 3, solid arrows).

### Electroeluted polypeptide bands

**Fig. 4:**
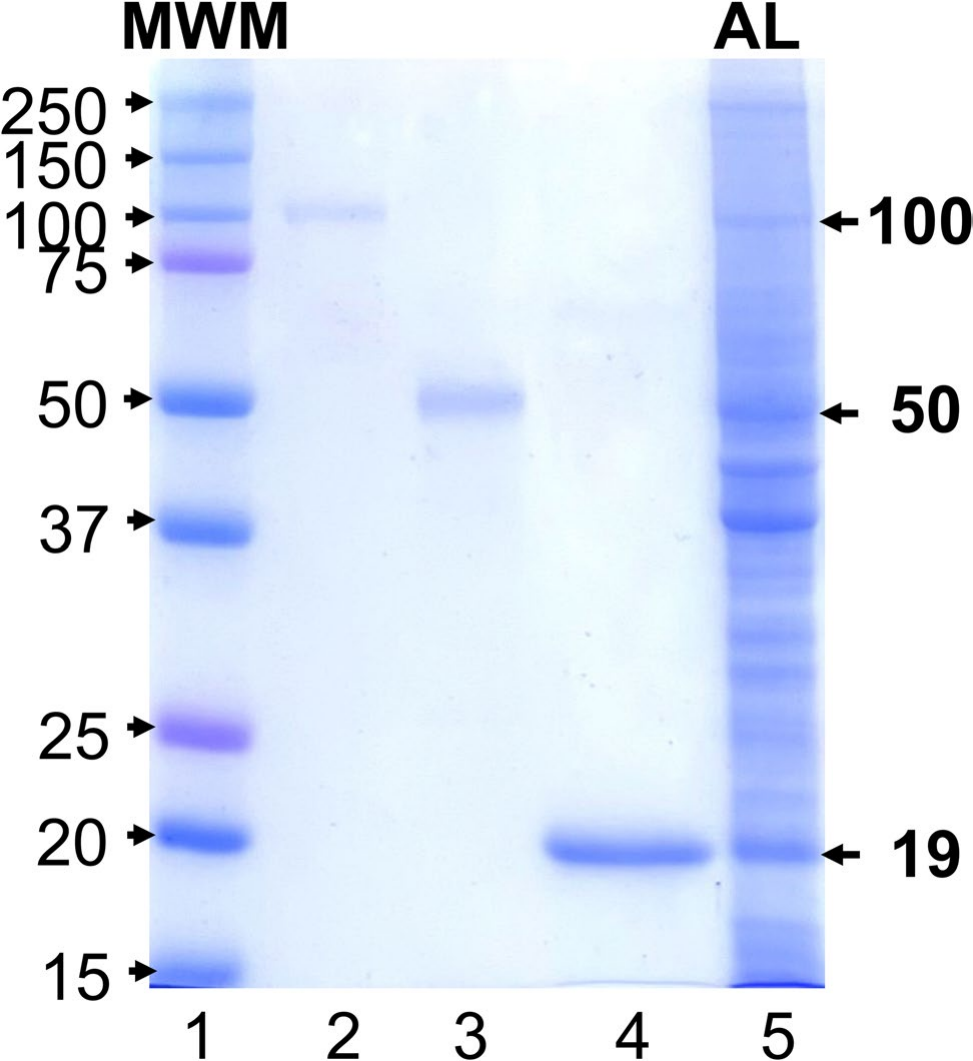
Electroeluted polypeptide bands. Polypeptide bands of 100, 50, and 19 kDa were identified, cut, and electroeluted from *N. fowleri* lysate. The polypeptide bands obtained were analyzed by the SDS-PAGE technique. (**1**) Molecular weight marker (MWM). (**2**) Electroeluted 100 kDa polypeptide band. (**3**) Electroeluted 50 kDa polypeptide band. (**4**) Electroeluted 19 kDa polypeptide band. (**5**) Amoebic lysate (AL). The numbers on the left and right represent the molecular weight in kDa

Due to the consistency and intensity in the recognition of some polypeptide bands of *N. fowleri* by the serum IgG and IgA isotypes of both residents of Mexicali and CDMX, the bands of 19, 50, and 100 kDa were electro-eluted and analyzed by SDS-PAGE techniques (Fig. 4). The molecular weight of the electro-eluted bands calculated with a standard protein marker (BioRad) was 19, 50, and 100 kDa (Fig. 4, lines 2, 3, and 4 respectively) and amoebic lysate as control (Fig. 4, lines 5).

### Recognition of electroeluted polypeptide bands by serum samples

**Fig. 5:**
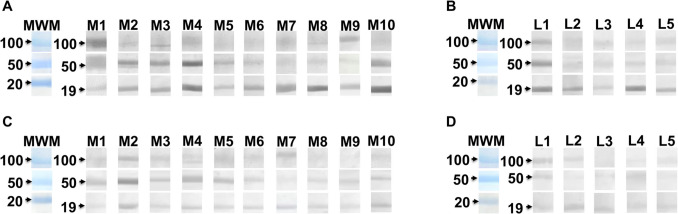
Recognition of immunogenic polypeptides by serum samples. Polypeptide bands were purified and electroeluted from *N. fowleri* lysate. They were subsequently transferred to nitrocellulose membranes. The membranes were incubated with serum from Mexicali Valley (**A** and **C**) or CDMX (**B** and **D**) residents. IgG (**A** and **B**) or IgA (**C** and **D**). The numbers on the left represent the molecular weight in kDa. Mexicali (M1–M10) and CDMX (L1–L5) samples. Antigens present in 100, 50, and 19 kDa polypeptide bands were recognized by both IgG and IgA isotypes. They showed different levels of recognition, with the IgG isotype showing the highest intensity in most of the samples evaluated compared to the IgA isotype

We also evaluated by a Western blot the recognition intensity given by the same antibodies towards the 19, 50, and 100 kDa polypeptides that had previously been electroeluted. Unlike what was reported with the entire immobilized *N. fowleri* extract, when we immobilized only the electroeluted bands, both IgG and IgA from all subjects were able to recognize the three polypeptide bands with different levels of intensity towards each band and each subject. (Fig. 5A–D).

We first observed the recognition patterns of serum IgG from the Mexicali group towards three specific polypeptide bands. The intensity of recognition varied among subjects. For instance, the IgG from subject M1 recognized all three polypeptide bands with significant intensity, particularly bands with molecular weights of 100 and 50 kDa. In contrast, the IgG from subjects M4 and M10 showed a stronger recognition of the 50 kDa polypeptide band. Additionally, the IgG from subjects M4, M7, M8, and M10 demonstrated a strong recognition of the 19 kDa polypeptide band (Fig. 5A).

In the analysis of IgA recognition among the Mexicali subjects, we observed that all subjects identified three polypeptide bands with varying intensities. Notably, the IgA from subjects M2 and M4 showed a strong recognition of the 50 kDa band, while the IgA from subject M2 also demonstrated high-intensity recognition of the 19 kDa band (Fig. 5C).

A similar pattern is observed in the recognition of both isotypes from the CDMX group samples. In this group, all subjects with immunoglobulins (IgG and IgA) recognize three bands. Notably, the IgG from subject number 1 (L1) shows strong recognition of all three polypeptide bands, while the IgG from subject L4, alongside L1, was particularly intense at recognizing the 19 kDa band (Fig. 5B). In contrast, the IgA from this same group shows a generally weaker recognition of the three polypeptide bands. However, it is worth mentioning that the IgA from subject L1 also shows strong recognition of all three polypeptide bands (Fig. 5D).

### Naegleria fowleri surface antigens are recognized by IgG serum samples

**Fig. 6:**
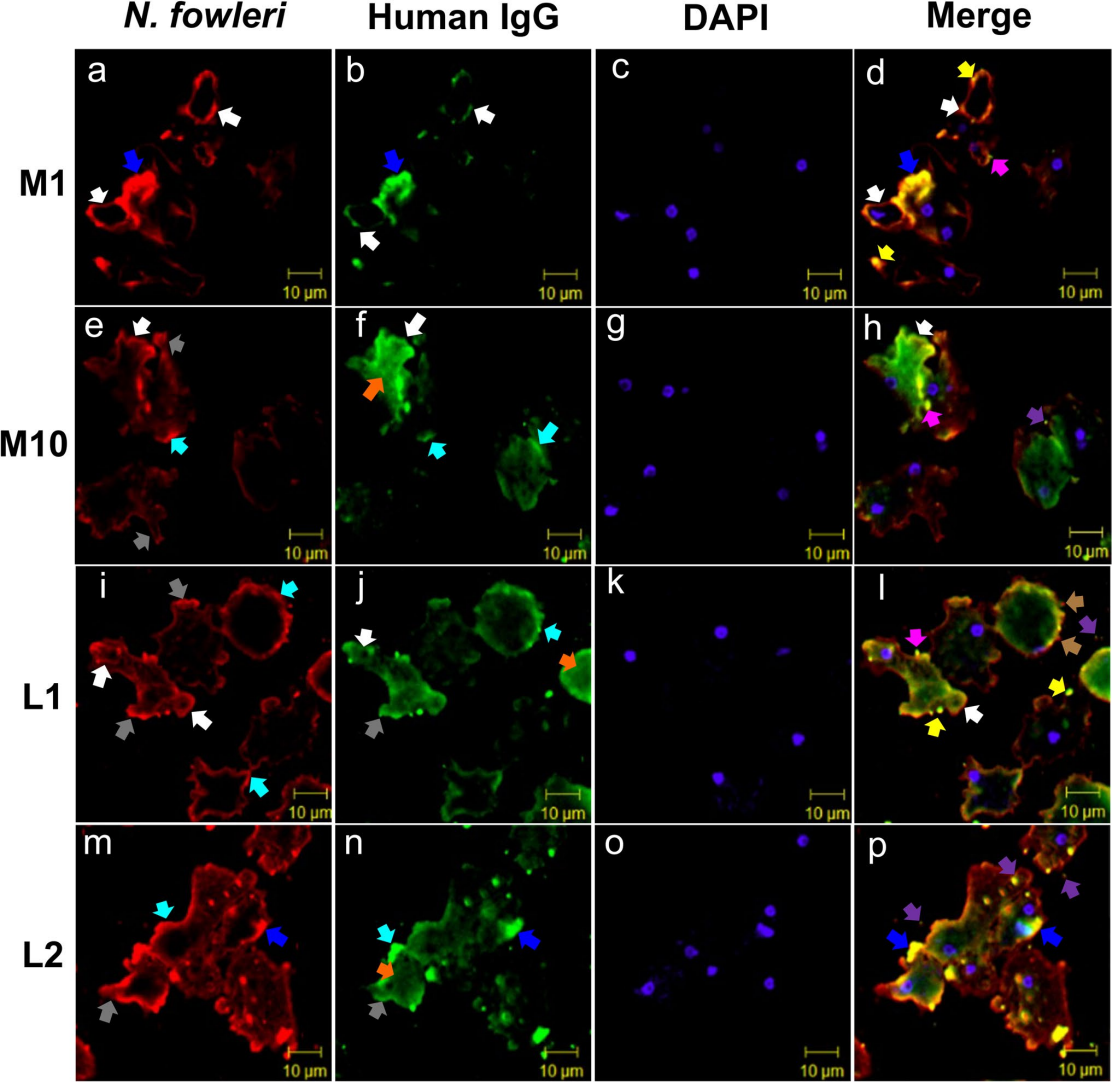
Recognition of *Naegleria fowleri* trophozoites by IgG antibodies from serum samples. *N. fowleri* trophozoites were detected by fluorescence immunocytochemistry and incubated with serum from Mexicali (Fig. 6, M1 and M10, **a**-**h**) or CDMX (Fig. 6, L1 and L2, **i**-**p**) residents. All samples were incubated with donkey anti-rabbit IgG Alexa Fluor 647 (red stain) or goat anti-human IgG, FITC (green stain). The color code of the arrows represents: white arrows, cytoplasmic extensions known as food cups recognized by both anti-*N. fowleri* and anti-human antibodies; turquoise arrows, regions of the trophozoite membrane in which an increase in recognition by both antibodies will be observed; gray arrows, cytoplasmic extensions known as pseudopodia with a positive label for both antibodies; orange arrows, recognition of cytoplasmic components mainly by anti-human antibodies; pink arrows, the colocalization of both antibodies in small vesicle-like in a process of detachment from the trophozoite membrane; blue arrows, presence of possible multivesicular bodies fusing with the cytoplasmic membrane showing the recognition of both antibodies; brown arrows, small vesicle-like recognized only by the anti-*N. fowleri* antibody in contact with the trophozoite membrane; yellow arrows; small vesicle-like molecules located in contact with the cytoplasmic membrane with the recognition of both antibodies and purple arrows, small vesicle-like molecules located in the extracellular medium with the recognition of both antibodies. Nuclei were visualized by DAPI staining (blue mark). Images were analyzed with an Axioscop 2 plus confocal fluorescence microscope (Carl Zeiss)

Due to the intensity of recognition given by both isotypes (IgG and IgA) from some samples observed by Western blot such as M1 and M10 from Mexicali and L1 and L2 from CDMX, both in *N. fowleri* lysate and electro-eluted polypeptide bands, we selected these samples to determine the distribution pattern given by the IgG antibodies of these subjects on *N. fowleri* trophozoites (Fig. 6).

Trophozoites were labelled with rabbit anti-*N-fowleri* antibodies (Fig. 6, red mark) where we can observe a membrane, pseudopodia, and food-cup recognition (Fig. 6, turquoise, gray, and white arrows respectively) while the IgG of the serum samples evaluated from both Mexicali and CDMX recognized different structures of *N. fowleri* trophozoites (Fig. 6, green mark). In this, in addition to the membrane, pseudopodia, and food cups, cytoplasm recognition was observed (orange arrows). Interestingly, although both antibodies recognized the same structures in both the Mexicali and CDMX samples, their distribution was not similar to M1 and M10 samples; both antibodies were observed in a more localized manner in food cups (Fig. 6a, b and e, f), while in L1 and L2 samples, the recognition of both antibodies was observed in a continuous manner throughout the membrane including pseudopodia and food cups (Fig. 6i, j and m, n). On the other hand, in L1 and L2 samples, a greater amount of small like-vesicle structures was observed compared to M1 and M10 samples recognized by one (merge, brown arrows) or both anti-*N. fowleri* and human IgG antibodies. Surprisingly, both labels (red and green) were co-localized in food cups and in small like-vesicles (Fig. 6, merge). These vesicles were observed detaching or in contact with the membrane surface (pink and yellow arrows respectively), in the process of secretion (blue arrows), or in the extracellular medium (purple arrows). Finally, in M1 and L2 samples, more antibody co-localization was observed compared to samples M10 and L1.

## Discussion

Recently in our working group, through morphological analysis and molecular sequencing, it was reported for the first time the presence of the genus Naegleria amoebae isolated from water collected from different irrigation channels in the Mexicali Valley. In that work, it was described not only the presence of non-pathogenic species such as *N. gruberi, N. clarki*, and *N. pagei* but also pathogenic species such as *N. fowleri* and *N. australiensis* (Bonilla-Lemus et al. 2020). Furthermore, in a previous work, the presence of *N. fowleri* in cerebrospinal fluid of patients (age range 1–18) with PAM who had swum or bathed particularly in an artificial channel of Mexicali Valley had previously been reported (Lares-Villa et al. 1993).

Although the majority of clinical cases for PAM in México have been reported in the Mexicali Valley (Cervantes-Sandoval et al. 2007; Lares-Villa et al. 1993; Valenzuela et al. 1984), coupled with the presence of the amoebae that has also been identified in the irrigation channels of the same valley (Lares-Villa et al. 1993; Valenzuela et al. 1984), to date, there is no serological study of the immunological response as well as the antigenic determinants that trigger this response in apparently healthy residents who occupy these irrigation channels to carry out recreational aquatic activities. Therefore, the present work focused on evaluating the levels of IgG and IgA antibodies as well as the antigenic recognition of IgG antibodies from serum samples obtained from residents of an area considered endemic such as the Mexicali Valley channels; on the other hand, it was also considered a group of people from an area considered non-endemic such as CDMX.

In general, all the subjects evaluated presented levels of specific antibodies against *N. fowleri*, being observed in the Mexicali samples the highest levels of both IgG and IgA (Fig. 2A). Furthermore, in both populations (Mexicali and CDMX), IgG was the isotype that presented the highest levels (*p* < 0.001***) with respect to IgA (Fig. 2B and C).

In previous studies, the immune response against *N. fowleri* had already been analyzed. For example, the titers of IgA antibodies in serum and saliva of individuals residing in Mexico who lived or not in endemic areas of this amoeba such as Ciudad Valles, San Luis Potosí, México, found that the recognition of *N. fowleri* proteins by serum and saliva IgA antibodies of people with upper respiratory tract infections (URTI) living in endemic areas were significantly higher compared to healthy individuals who are residents of the same area (Rivera et al. 2001). On the other hand, it has been reported that IgG antibodies in serum samples from healthy children and adolescents living in Potam, Vicam, and Cócorit, three villages of Sonora, México, as well as from patients admitted to a Regional Medical Center, recognize proteins from *Naegleria fowleri*, *N. lovaniensis*, *Balamuthia mandrillaris*, and *Acanthamoeba* sp. T4 (Dubray et al. 1987; Lares-Jiménez et al. 2018).

Despite various studies conducted on this topic, no correlation has been established between susceptibility to primary amebic meningoencephalitis (PAM) and humoral immune status, specifically the levels and specific responses of IgA or IgG antibodies against *Naegleria fowleri*. For instance, serum samples from diverse populations have tested positive for antigenic material from *Naegleria* species. This includes samples from individuals who experienced “wet fever” (Finnegan et al. 1987), randomly collected serum from hospitalized patients (Dubray et al. 1987), and samples from army recruits in the USA with acute respiratory diseases (Powell et al. 1994). Additionally, seropositive samples were found among healthy individuals in New Zealand (Cursons et al. 1980) and Czechoslovakia (Cerva 1989), indicating the presence of antibodies against some free-living amoebae, including *N. fowleri.*

Therefore, because almost all serum from healthy individuals has been positive for *N. fowleri*, it can be assumed that amoebae exposure is common. The fact that in the present work we found levels of both IgG and IgA in Mexicali samples may suggest a constant stimulation of the immune response with *N. fowleri* antigens causing an immunoprotective response against this amoeba of people residing in this place or who have contact with such channels. We do not discard that the inhibition of the *N. fowleri* trophozoite adhesion to nasal epithelium mediated by specific IgG and IgA antibodies at the lumen level may be crucial to induce protection against PAM in inhabitants of areas endemic for this amoeba such as Mexicali Valley channels. In the murine experimental model, we have reported that intranasal administration of *N. fowleri* lysate co-administered with cholera toxin increases protection against meningoencephalitis caused by *N. fowleri* in immunized and challenged mice (Carrasco-Yepez et al. 2014; Rojas-Hernández et al. 2020). In these works, an increase in the levels of both IgA and IgG in serum and nasal washes has been found. This response has also been found at the lumen level where the antibodies could be preventing the adhesion of the pathogen to the nasal epithelium of the mouse. As this has been mentioned, these antibodies are not limited to detection in nasal mucosa but have also been shown in plasma. Particularly systemic IgG from protected immunized mice strongly recognize *N. fowleri* immunogenic antigens (Rojas-Hernández et al. 2020). Therefore, the predominance of systemic IgG found in both groups of subjects as well as the intense recognition of this antibody towards specific antigens of *N. fowleri*, particularly in the Mexicali group, could be associated with protection against PAM in possible contact with the amoebae.

Studies examining human serum and mucosal antibody levels have found widespread evidence of immune responses against *N. fowleri* resulting from subclinical exposure to the organism (Dubray et al. 1987; Lares-Jiménez et al. 2018; Rivera et al. 2001). These immune responses may arise after a nonolfactory exposure or olfactory clearance of less pathogenic strains of *N. fowleri.* There is no evidence to support the idea that overt immunodeficiency increases the risk of *N. fowleri* infection. Instead, the presence of varying but detectable immune responses indicates that differences in both innate and adaptive immunity play a significant role in the development of primary amoebic meningoencephalitis (PAM) (Moseman 2020). It has been suggested that antibodies against certain parasites could be the result of exogenous stimulation of antigens of bacterial, viral, or plant origin (Ferrante & Allison 1983). Antibodies that recognize *N. fowleri* proteins can be induced by specific immunizations or cross-reactions by infections with other genera and species of amoebae (Rivera et al. 2001). For example, it has been shown that human serum samples from random hospitalized patients have close similarity in serological results to detect antibodies to *N. fowleri* and *N. lovaniensis* by using western blot assay (Dubray et al. 1987). As we mentioned previously, we have identified the presence of non-pathogenic species such as *N. gruberi*, *N. clarki*, and *N. pagei* in Mexicali Valley channels to which residents may be exposed and generate immunological responses capable of recognizing antigens shared with *N. fowleri* (Bonilla-Lemus et al. 2020). There is a phylogenetic relationship among *Naegleria* spp., as previous studies have shown that there are highly conserved genetic regions, both in coding and non-coding areas, across different species of Naegleria (Aurongzeb et al. 2025; Bonilla-Lemus et al. 2020; Joseph et al. 2021; Montalbano Di Filippo et al. 2017). Additionally, it has been reported that certain virulence factors are not exclusive to *N. fowleri*; some have also been identified in non-pathogenic Naegleria species, albeit with varying levels of expression or post-translational modifications (Gutiérrez-Sánchez et al. 2020). Finally, the recognition of certain antigens by antibodies from CDMX individuals may be linked to the presence of different strains or species of non-pathogenic free-living that are antigenically related to *N. fowleri.* It is important to mention that the potential role of cross-reactive epitopes in *Naegleria* spp. antigens can lead to the development of antibodies in individuals living in areas endemic to this amoeba. Following exposure to different species found in the Mexicali channels, cross-reactive B cells may undergo somatic hypermutation, which enhances their affinity and specificity for shared or similar antigens. This polyreactivity could evolve into a monospecificity of certain antibodies for specific epitopes of *N. fowleri*, such as those contained in the polypeptide bands of 100, 50, or 19 kDa. This process could ultimately provide protection against PAM after multiple exposures to non-pathogenic Naegleria species within the endemic population.

Due to the detectable but variable immune response that we observed in all samples from each subject, we decided to evaluate the polypeptide bands molecular weight of *N. fowleri* that could be triggering this immune response. Both IgG and IgA antibodies from serum samples of residents from Mexicali and CDMX recognized proteins from *N. fowleri* at various molecular weights. Some polypeptide bands were recognized more intensely than others. Notably, the antigenic recognition pattern in residents of Mexicali was stronger and more diverse compared to that of residents from CDMX. The recognition of several antigens mainly by serum IgG from Mexicali residents observed in this study allows us to suggest that these polypeptide bands present highly immunogenic antigenic determinants which are probably responsible for the levels of both IgG and IgA antibodies observed in this study (Fig. 2).

On the other hand, the antigenic recognition by both the IgG and IgA isotypes observed in this study coincided mainly in the polypeptide bands of 100, 50, and 19 kDa in both populations evaluated, the same immunogenic bands that we had previously reported in the experimental model of PAM in mice where IgG and IgA antibody response progressively augmented in relation to the increase of number of immunizations with *N. fowleri* lysate plus cholera toxin and that this response was directed mainly towards the polypeptides of 250, 100, 70, 50, 37, and 19 kDa (Rojas-Hernández et al. 2020). Furthermore, most of these polypeptide bands are differentially expressed in *N. fowleri* compared to *N. lovaniensis* (non-pathogenic species) (Gutiérrez-Sánchez et al. 2020).

Through mass spectrometry, we have reported that the 100 and 50 kDa polypeptides have fragments of actin, membrane proteins, myosin, and Hsp70 among others, while the 19 kDa protein band has mainly membrane protein MP2CL5 (Gutiérrez-Sánchez et al. 2020; Rojas-Hernández et al. 2020). It is important to mention that the MP2CL5 protein has been reported in *N. fowleri*, but it is absent in non-pathogenic species suggesting that this protein is an important virulence factor (Réveiller et al. 2001).

Due to certain polypeptide bands exhibiting strong reactivity with most sera from different subjects, these bands (100, 50, and 19 kDa) were purified and evaluated separately. Both IgG and IgA from all subjects recognized the three electroeluted polypeptides at different intensities; however, Mexicali serum samples showed the greatest immunogenicity. Western blot analysis shows the reactivity of the IgA e IgG serum antibodies of all subjects with the electroeluted polypeptide bands of *N. fowleri*. This result could be due to the exposure of the antigenic determinants after the electrophoretic shift as well as the specificity of the antibodies which are interacting with only a purified band in comparison to the approximately 70 bands present in the total extract. The purified polypeptide band might have repeated antigen sites that exhibit determinants that promote antibody binding (Sela-Culang et al. 2013). Different aspects could alter or affect the antigenic properties of the polypeptide bands when they are electroeluted. As a result, this process may uncover certain linear epitopes that are preferentially recognized by the antibodies found in the subjects studied, compared to the total extract of *N. fowleri*.

Taken together, these results and those obtained in the present work, we suggest that these polypeptide bands can be considered possible candidates for vaccine development since these polypeptide bands are not only strongly recognized by mice antibodies that have been immunized and protected against PAM, but also by antibodies from individuals who may have been directly exposed to antigens from *N. fowleri* or other similar amoebae and consequently generate a cross-reactivity antibody response.

Finally, we also decided to evaluate the serum antibody recognition pattern of some subjects towards fixed trophozoites of *N. fowleri*. Interestingly, although rabbit anti-*N. fowleri* antibodies as well as IgG antibodies from serum samples mainly recognized the membrane, pseudopodia, food cups, and small like-vesicles, the distribution was not similar. The presence of IgG antibodies in serum, concentrated in food cups, highlights the significance of the antigens in these structures. Additionally, the similarity with the recognition of rabbit antibodies indicates that individuals from Mexicali are likely exposed to continuous immunization. Previously, we mentioned that among the most immunogenic polypeptides were membrane proteins as well as myosin and actin which have been found distributed mainly in pseudopodia and food cups (Diakonova et al. 2002; Sohn, Kim, Shin, Song, & Shin, 2010; Zysset-Burri et al. 2014). Therefore, it is interesting to show that antibody subjects could be recognizing these trophozoite structures. Surprisingly, small like-vesicles distributed throughout the cytoplasm, in the trophozoites membrane, or located in the extracellular environment were also recognized by these antibodies. In this sense, in previous studies, we have observed the secretion of vesicles in the nasal cavity of mice infected with *N. fowleri* trophozoites. In a study where we evaluated the ability of *N. fowleri* to induce NET release from PMN cells in mice in vitro and in a murine model of PAM, they showed that *N. fowleri* induces both NET and MPO released from PMN cells in mice after exposure to trophozoites in a time-dependent manner suggesting that NETs are somehow associated with amoebae (M. M. Carrasco-Yepez et al. 2019). In another work where we evaluated the presence, expression, and function of cathepsin B of *N. fowleri* in vitro and during PAM, we reported the secretion of vesicles by trophozoites after interaction with PMN as well as in the nasal cavity of infected mice (Rodríguez-Mera et al. 2022). Interestingly, in both studies, vesicle secretion was observed, leading us to suggest that in this study, recognition of vesicles by serum IgG antibodies may be due to interaction with *N. fowleri* or antigenically similar amoebae that produced a recognition of antigens present in the vesicles.

Although we cannot affirm specifically which proteins were recognized in the small like-vesicle by the antibodies present in the serum samples evaluated, we can suggest their possible participation in the infectious process, as well as the induction of immunoprotective responses in people exposed directly or indirectly to *N. fowleri*. However, the role of vesicles as well as the antigens recognized by antibodies remains to be studied. Finally, the differences observed in the fixed trophozoites suggest a heterogeneous culture; that is, the amoebas are carrying out different activities.

## Conclusion

The results indicate that the immune response, as detected by levels of IgG and IgA antibodies and the recognition of specific immunogenic antigens might be important for protecting against the infectious process of PAM. The 100, 50, and 19 kDa polypeptides may serve as potential therapeutic targets or candidates for vaccine design against *N. fowleri* infection. However, further research is required to validate the recognition of these antigens in broader populations. It is important to thoroughly analyze their relevance for immunoepidemiological studies. Unfortunately, the lack of aquatic recreational facilities in the Mexicali area, coupled with many zones with low socioeconomic conditions and warm, semi-arid environmental characteristics, leads people to continue exposing themselves to channels where they swim, especially on hot days (spring to autumn). This behavior increases the risk of contact with *N. fowleri*, despite the known dangers associated with this amoeba.

## Supplementary Information

Below is the link to the electronic supplementary material.

Supplementary file1 (PDF 5631 KB)

## Data Availability

No datasets were generated or analysed during the current study.
