# Supplementary material for: Identification of Naegleria fowleri antigens recognized by serum antibodies from people of Mexicali Valley, México
Source: Parasitol Res. 2025 Mar 15;124(3):33. doi: 10.1007/s00436-025-08476-2 (PMC11910403; doi:10.1007/s00436-025-08476-2)
Supplement: Supplementary file 1 — Supplementary file1 (PDF 5631 KB) [file 436_2025_8476_MOESM1_ESM.pdf]

Figure 3A in the manuscript.
Nitrocellulose membrane for Western blot analysis
Recognition of *N. fowleri* polypeptide bands by IgG antibodies from Mexicali samples.

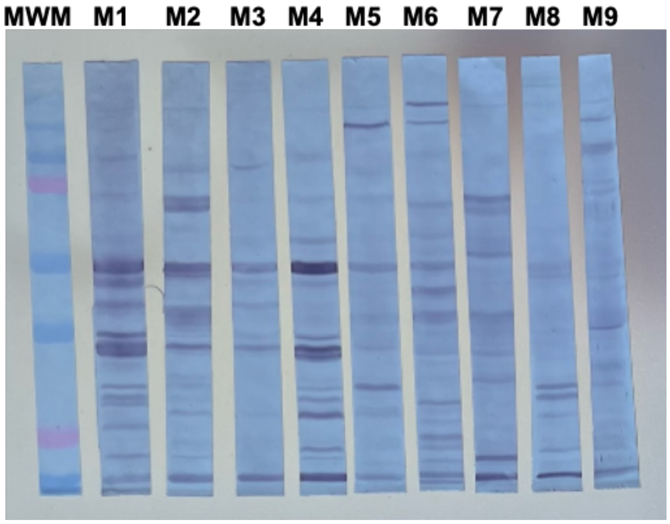

N. fowleri lysates were subjected to SDS-PAGE electrophoresis and transferred to nitrocellulose membranes. The membranes were incubated with serum from Mexicali Valley residents. Then, Goat anti-Human IgG HRP was added.

Figure 3B in the manuscript.
Nitrocellulose membrane for Western blot analysis
Recognition of *N. fowleri* polypeptide bands by IgG antibodies from CDMX samples.

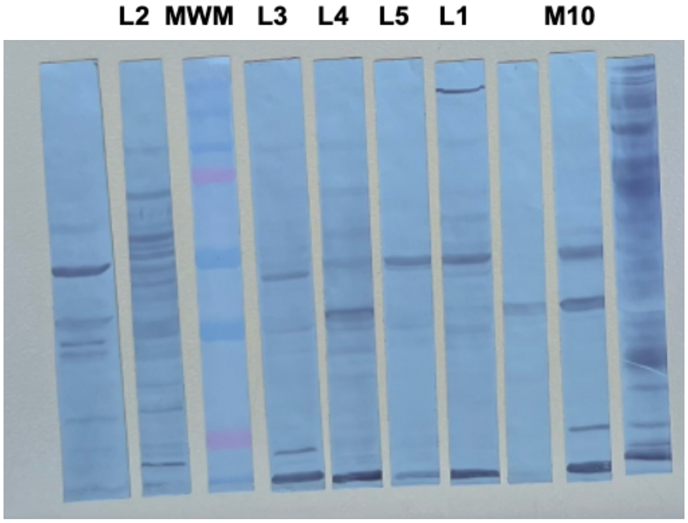

N. fowleri lysates were subjected to SDS-PAGE electrophoresis and transferred to nitrocellulose membranes. The membranes were incubated with serum from CDMX residents. Then, Goat anti-Human IgG HRP was added.

Figure 3C in the manuscript.
Nitrocellulose membrane for Western blot analysis
Recognition of *N. fowleri* polypeptide bands by IgA antibodies from Mexicali samples.

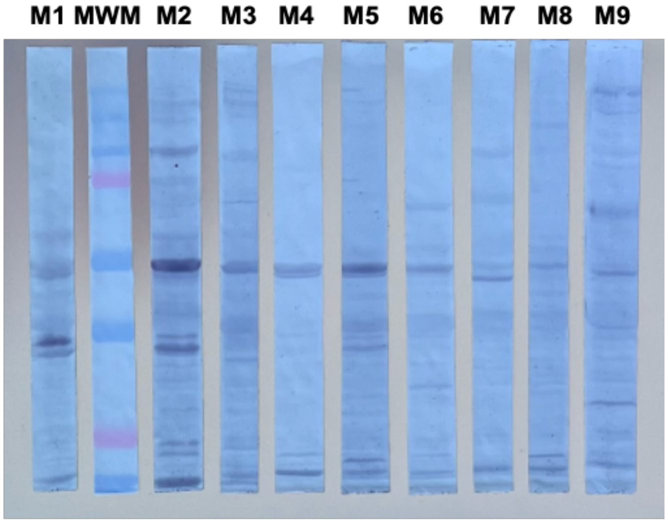

N. fowleri lysates were subjected to SDS-PAGE electrophoresis and transferred to nitrocellulose membranes. The membranes were incubated with serum from Mexicali Valley residents. Then, Goat anti-Human IgA HRP was added.

Figure 3D in the manuscript.
Nitrocellulose membrane for Western blot analysis
Recognition of *N. fowleri* polypeptide bands by IgA antibodies from CDMX samples.

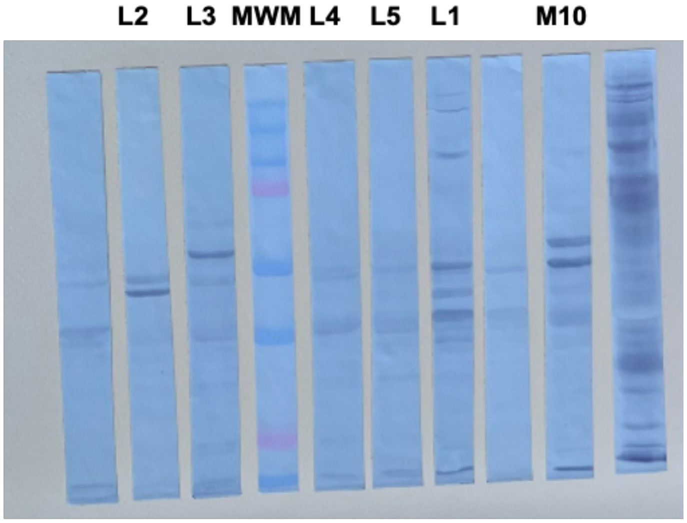

N. fowleri lysates were subjected to SDS-PAGE electrophoresis and transferred to nitrocellulose membranes. The membranes were incubated with serum from CDMX residents. Then, Goat anti-Human IgA HRP was added.

Figure 4 in the manuscript.
Polyacrylamide gel stained with Coomassie blue

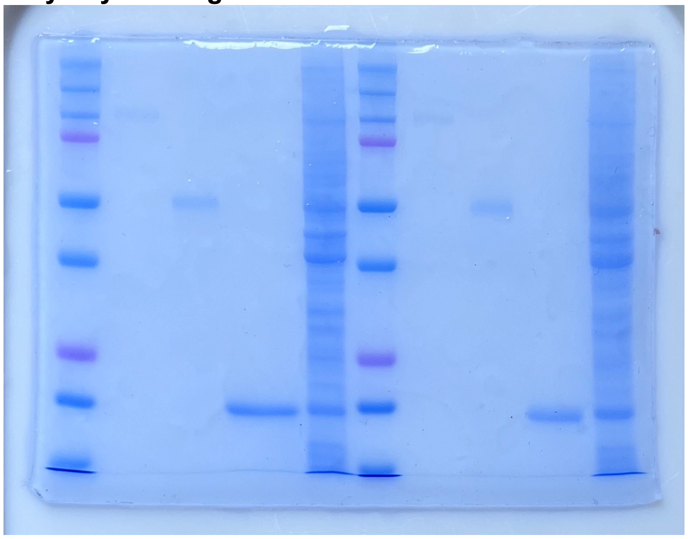

Polypeptide bands of 100, 50 and 19 kDa were identified, cut and electroeluted from *N. fowleri* lysate. The polypeptide bands obtained were analyzed by SDS-PAGE technique. (1) Molecular weight marker (MWM). (2) Electroeluted 100 kDa polypeptide band. (3) Electroeluted 50 kDa polypeptide band. (4) Electroeluted 19 kDa polypeptide band. (5) Amoebic lysate (AL).

Figure 5A in the manuscript.
Nitrocellulose membrane for Western blot analysis
Recognition of immunogenic polypeptides by IgG antibodies from Mexicali samples.

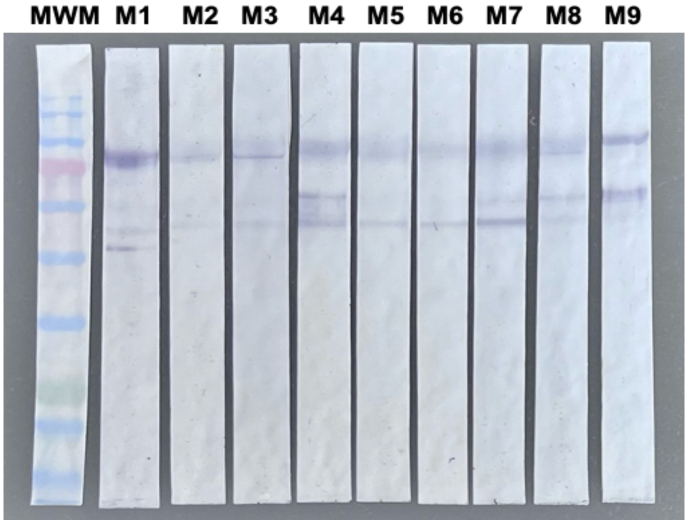

Polypeptide bands were purified and electroeluted from *N. fowleri* lysate. They were subsequently transferred to nitrocellulose membranes. The membranes were incubated with serum from Mexicali Valley residents. Then, Goat anti-Human IgG HRP was added.

Figure 5B in the manuscript.
Nitrocellulose membrane for Western blot analysis
Recognition of immunogenic polypeptides by IgG antibodies from CDMX samples.

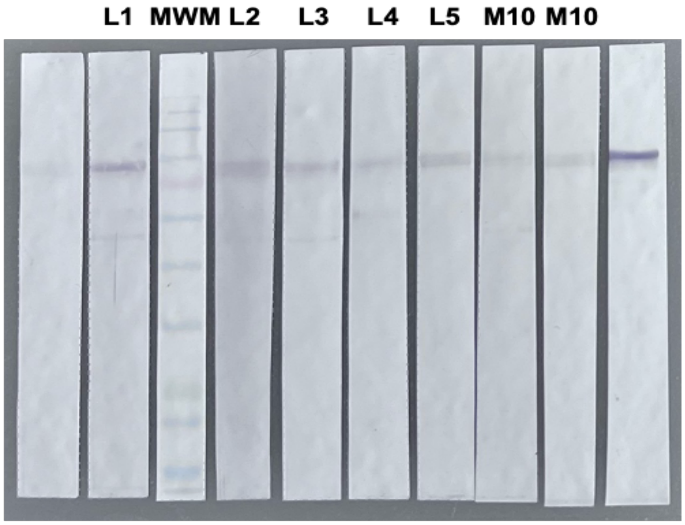

Polypeptide bands were purified and electroeluted from *N. fowleri* lysate. They were subsequently transferred to nitrocellulose membranes. The membranes were incubated with serum from CDMX residents. Then, Goat anti-Human IgG HRP was added.

Figure 5C in the manuscript.
Nitrocellulose membrane for Western blot analysis
Recognition of immunogenic polypeptides by IgA antibodies from Mexicali samples.

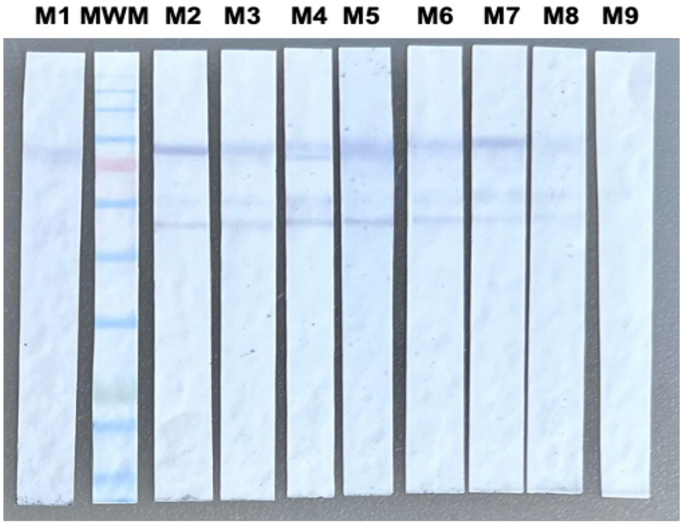

Polypeptide bands were purified and electroeluted from *N. fowleri* lysate. They were subsequently transferred to nitrocellulose membranes. The membranes were incubated with serum from Mexicali Valley residents. Then, Goat anti-Human IgA HRP was added.

Figure 5D in the manuscript.
Nitrocellulose membrane for Western blot analysis IgA CDMX
Recognition of immunogenic polypeptides by IgA antibodies from CDMX samples.

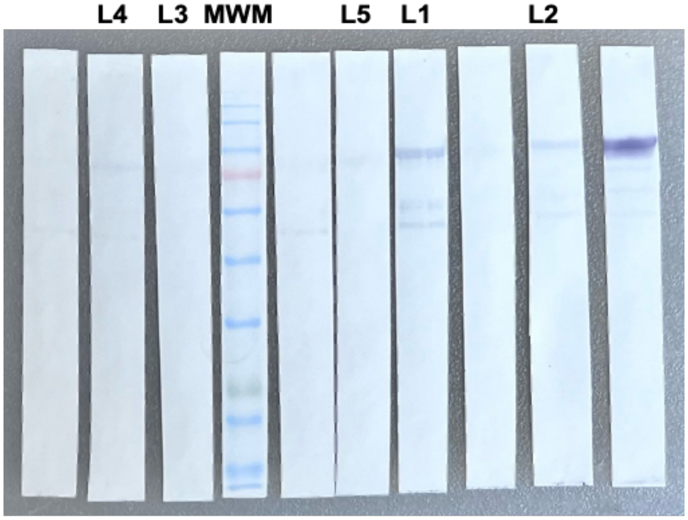

Polypeptide bands were purified and electroeluted from *N. fowleri* lysate. They were subsequently transferred to nitrocellulose membranes. The membranes were incubated with serum from CDMX residents. Then, Goat anti-Human IgA HRP was added.

Figure 5A in the manuscript.
Nitrocellulose membrane for Western blot analysis
Recognition of immunogenic polypeptides by IgG antibodies from Mexicali samples.

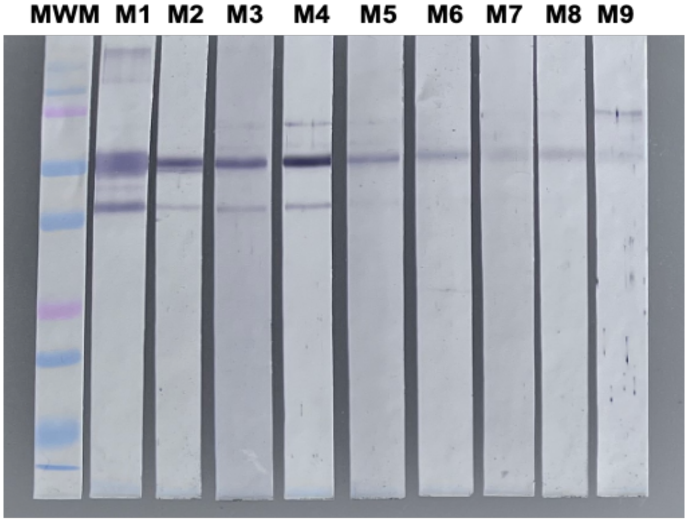

Polypeptide bands were purified and electroeluted from *N. fowleri* lysate. They were subsequently transferred to nitrocellulose membranes. The membranes were incubated with serum from Mexicali Valley residents. Then, Goat anti-Human IgG HRP was added.

Figure 5B in the manuscript.
Nitrocellulose membrane for Western blot analysis
Recognition of immunogenic polypeptides by IgG antibodies from CDMX samples.

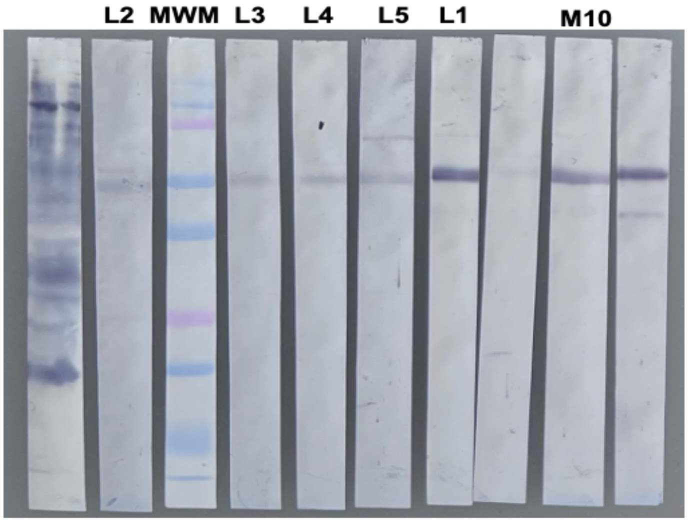

Polypeptide bands were purified and electroeluted from *N. fowleri* lysate. They were subsequently transferred to nitrocellulose membranes. The membranes were incubated with serum from CDMX residents. Then, Goat anti-Human IgG HRP was added.

Figure 5C in the manuscript.
Nitrocellulose membrane for Western blot analysis
Recognition of immunogenic polypeptides by IgA antibodies from Mexicali samples.

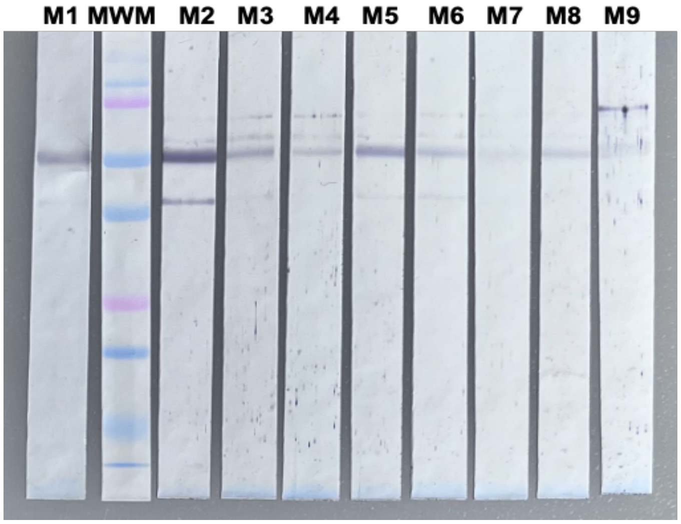

Polypeptide bands were purified and electroeluted from *N. fowleri* lysate. They were subsequently transferred to nitrocellulose membranes. The membranes were incubated with serum from Mexicali Valley residents. Then, Goat anti-Human IgA HRP was added.

Figure 5D in the manuscript.
Nitrocellulose membrane for Western blot analysis
Recognition of immunogenic polypeptides by IgA antibodies from CDMX samples.

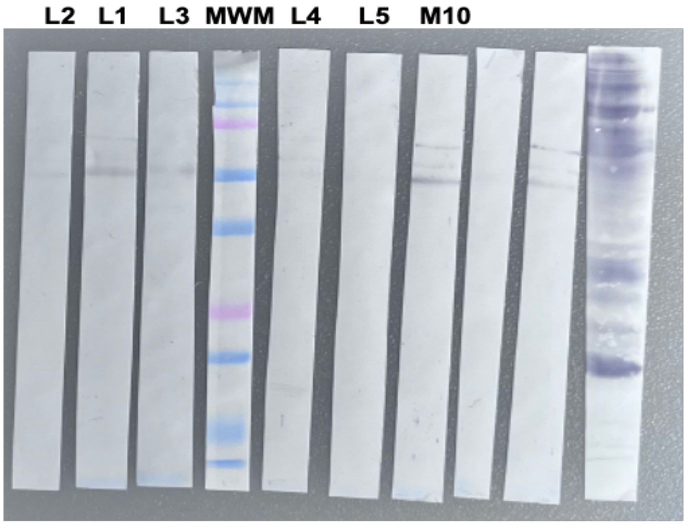

Polypeptide bands were purified and electroeluted from *N. fowleri* lysate. They were subsequently transferred to nitrocellulose membranes. The membranes were incubated with serum from CDMX residents. Then, Goat anti-Human IgA HRP was added.

Figure 5A in the manuscript.
Nitrocellulose membrane for Western blot analysis
Recognition of immunogenic polypeptides by IgG antibodies from Mexicali samples.

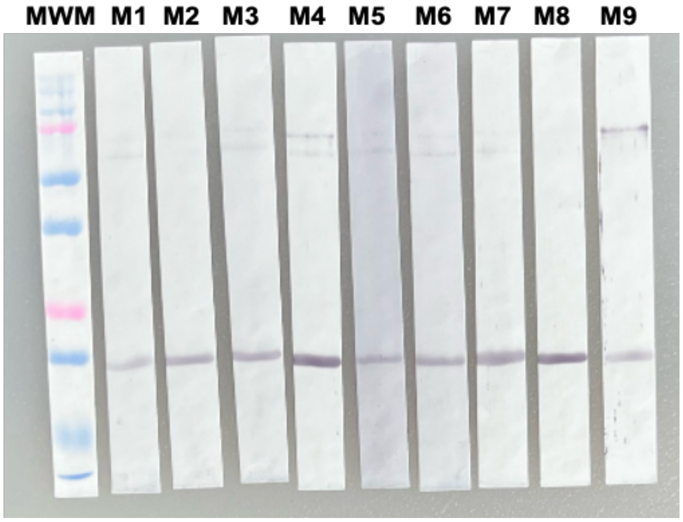

Polypeptide bands were purified and electroeluted from *N. fowleri* lysate. They were subsequently transferred to nitrocellulose membranes. The membranes were incubated with serum from Mexicali Valley residents. Then, Goat anti-Human IgG HRP was added.

Figure 5B in the manuscript.
Nitrocellulose membrane for Western blot analysis
Recognition of immunogenic polypeptides by IgG antibodies from CDMX samples.

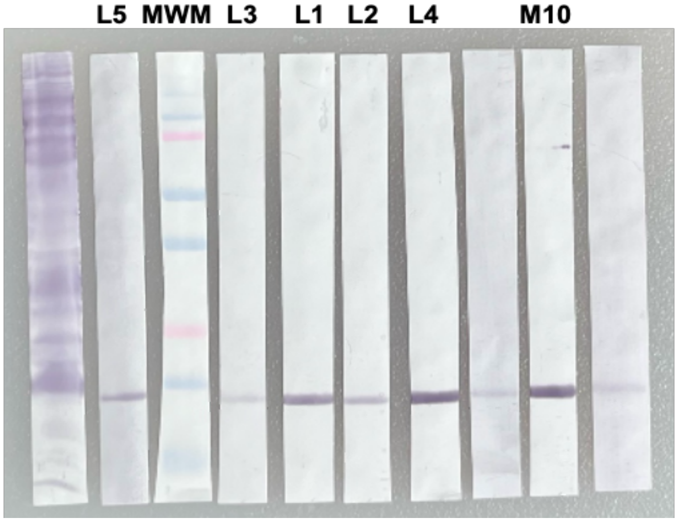

Polypeptide bands were purified and electroeluted from *N. fowleri* lysate. They were subsequently transferred to nitrocellulose membranes. The membranes were incubated with serum from CDMX residents. Then, Goat anti-Human IgG HRP was added.

Figure 5C in the manuscript.
Nitrocellulose membrane for Western blot analysis
Recognition of immunogenic polypeptides by IgA antibodies from Mexicali samples.

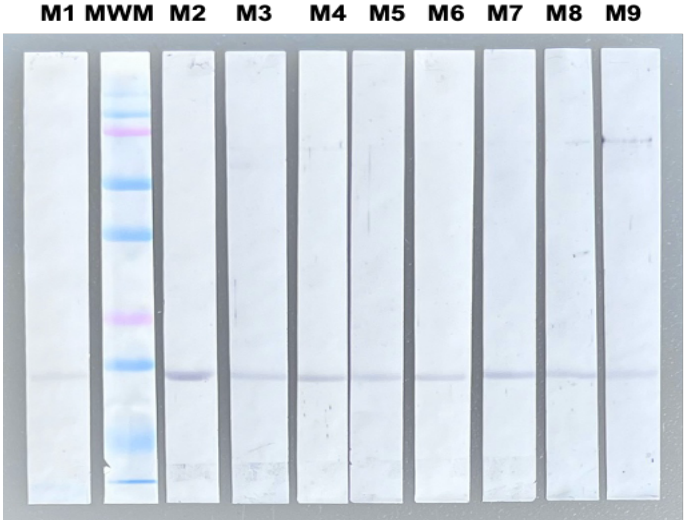

Polypeptide bands were purified and electroeluted from *N. fowleri* lysate. They were subsequently transferred to nitrocellulose membranes. The membranes were incubated with serum from Mexicali Valley residents. Then, Goat anti-Human IgA HRP was added.

Figure 5D in the manuscript.
Nitrocellulose membrane for Western blot analysis
Recognition of immunogenic polypeptides by IgA antibodies from CDMX samples.

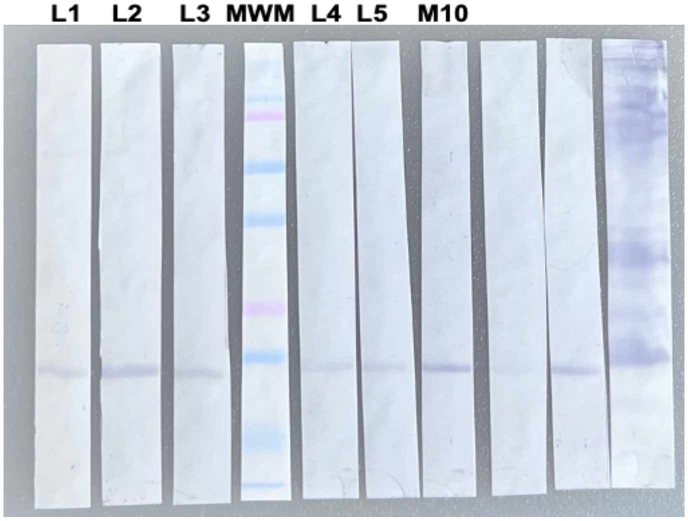

Polypeptide bands were purified and electroeluted from *N. fowleri* lysate. They were subsequently transferred to nitrocellulose membranes. The membranes were incubated with serum from CDMX residents. Then, Goat anti-Human IgA HRP was added.
